# The Hospital Conference in Cork

**Published:** 1911-07-08

**Authors:** 


### THE HOSPITAL CONFERENCE IN CORK.

Acting on the resolution passed at the last board meeting of the Trustees of the North Infirmary, a conference of representatives from the various city hospitals was held on June 26 to consider how the Insurance Bill would affect Irish institutions.

The following attended:—From the North Infirmary, Sir D. J. Hegarty, Rev. Dr. Hearn, Mr. Albert Cagney, Mr. D. H. Forde, and Mr. Stephen Perry, J.P.; from the South Infirmary, Very Rev. Canon McNamara, P.P., Mr. Samuel H. Newsom, and Mr. J. C. McNamara; from the Mercy Hospital, Rev. Father Keily, C.C., Mr. J. J. Horgan, solicitor, and Dr. Cullinane; from the Fever Hospital, Mr. M. D. Daly, J.P., Sir J. Harley Scott, J.P., and Dr. Sutton; from the Victoria Hospital, Mr. L. A. Beamish, J.P.; from the District Hospital, Mr. McCarthy (Chairman Board of Guardians) and Mr. J. J. Goggin, T.C.; from the Eye, Ear and Throat Hospital, Dr. Sandford; from the Protestant Incurable Hospital, Colonel Lunham, J.P., C.B. Mr. Maurice Healy, M.P., was also present.

The chair was taken by Mr. Samuel H. Newsom, on the motion of Mr. L. A. Beamish, seconded by Sir D. J. Hegarty. At the request of the meeting Mr. Perry acted as hon. secretary.

The Chairman said the object of their coming together was to consider how the proposed legislation would affect the future working of their hospitals. He feared they would financially suffer. In support of this he read copies of resolutions that had been passed in London and elsewhere on the subject, and extracts from some of the leading journals. They all felt grateful to Mr. Maurice Healy for coming to their assistance and for his presence there that day. He asked Mr. Forde, who had started the subject, to explain his views.

Mr. Forde, North Infirmary, feared that when employers found the large amount they would have to pay for the insurance of their employees they might be induced to lessen their contributions. Ireland was principally agricultural, and as a rule their people were healthy and lived long. There should certainly be a large surplus in the hands of the Government. He mentioned the matter in Mr. Healy's presence, feeling certain he would watch the various clauses in the Bill and their application to the public hospitals of the country.

Mr. Maurice Healy, M.P., then discussed several clauses of the Bill, pointing out that in reality the only hospitals taken into consideration in it were sanatoria where tuberculosis in all its stages was treated. He suggested passing some general resolution that day, and having their Secretary communicate with some of the leading hospitals here and in England, so that united action could be taken. All the hospitals in the three kingdoms would be equally affected by the Bill.

Rev. Dr. Hearn quite approved of Mr. Healy's suggestion, as did Mr. M. D. Daly, Mr. L. A. Beamish, Dr. Sandford, the Chairman, and Mr. J. J. Horgan. Ultimately the following resolution was proposed by Mr. L. A. Beamish, and seconded by Sir Daniel Hegarty, and passed unanimously :—" That this meeting is of opinion that joint action on the part of the hospitals of Ireland should be taken immediately to bring under the notice of the Chancellor of the Exchequer the injury likely to be inflicted on Irish hospitals unless the provisions of the Insurance Bill be modified as regards these institutions, and that we express our willingness to act with any central body in Ireland which is prepared to take this subject up."

Mr. M. D. Daly proposed, and Colonel Lunham seconded, and it was passed : " That the following Sub-committee be appointed to represent Cork City and County Hospitals and to communicate with the governing bodies of the principal Irish hospitals :—D. H. Forde, S. H. Newsom, Rev. Father Keily, Dr. Sandford, L. A. Beamish, M. D. Daly, J.P., Rev. F. X. McCarthy, Colonel Lunham, and Stephens Perry, J.P., hon. secretary."

The meeting was then adjourned to a future day, when the Sub-committee would have a report to submit. In the meantime an account of the proceedings of the conference has been sent, we understand, to every Irish hospital.

